# Distinct Levels of Reactive Oxygen Species Coordinate Metabolic Activity with Beta-cell Mass Plasticity

**DOI:** 10.1038/s41598-017-03873-9

**Published:** 2017-06-26

**Authors:** Ezzaldin Ahmed Alfar, Dilyana Kirova, Judith Konantz, Sarah Birke, Jörg Mansfeld, Nikolay Ninov

**Affiliations:** 1 DFG Center for Regenerative Therapies Dresden, Technische Universität Dresden, Dresden, Germany; 2 Paul Langerhans Institute Dresden of the Helmholtz Zentrum München at the University Hospital and Faculty of Medicine Carl Gustav Carus of Technische Universität Dresden, Dresden, Germany; 3 German Center for Diabetes Research (DZD e.V.), Neuherberg, Neuherberg, Germany; 4 Department of Pharmacology and Toxicology, Technische Universität Dresden, Dresden, Germany; 5 Cell Cycle, Biotechnology Center, Technische Universität Dresden, 01307 Dresden, Germany

## Abstract

The pancreatic beta-cells control glucose homeostasis by secreting insulin in response to nutrient intake. The number of beta-cells is under tight metabolic control, as this number increases with higher nutrient intake. However, the signaling pathways matching nutrition with beta-cell mass plasticity remain poorly defined. By applying pharmacological and genetic manipulations, we show that reactive oxygen species (ROS) regulate dose-dependently beta-cell proliferation *in vivo* and *in vitro*. In particular, reducing ROS levels in beta-cells blocks their proliferation in response to nutrients. Using a non-invasive genetic sensor of intracellular hydrogen peroxide (H_2_O_2_), we reveal that glucose can directly increase the levels of H_2_O_2_. Furthermore, a moderate increase in H_2_O_2_ levels can stimulate beta-cell proliferation. Interestingly, while high H_2_O_2_ levels are inhibitory to beta-cell proliferation, they expand beta-cell mass *in vivo* by inducing rapid beta-cell neogenesis. Our study thus reveals a ROS-level-dependent mechanism linking nutrients with beta-cell mass plasticity. Hence, given the requirement of ROS for beta-cell mass expansion, antioxidant therapies should be applied with caution in diabetes.

## Introduction

Diabetes is a metabolic disorder marked by chronic hyperglycemia. There are two major types of diabetes. Type one diabetes (T1D) is associated with an increase in blood glucose levels due to chronic autoimmune destruction of the beta-cells of the pancreas^1^. Type two diabetes (T2D) is characterized by an increase in the peripheral demand for insulin due to insulin resistance. In the initial phases of T2D, beta-cells can compensate for the increased insulin demand by expansion and/or by producing more insulin. However, as the disease progresses, the capacity of beta-cells to compensate for the increased insulin demand is reduced due to an increase in cellular stress and beta-cell loss^2^.

Among the factors that contribute to beta-cell dysfunction in diabetes, oxidative stress is thought to play an important role^3^. Notably, beta-cells express very low levels of antioxidant enzymes, such as catalase and superoxide-dismutase, which make them susceptible to oxidative damage^4, 5^. Indeed, oxidative stress is frequently observed in islets of both human diabetes patients and mouse models of insulin resistance^6–8^. Additionally, treatment of beta-cells *in vitro* with hydrogen peroxide (H_2_O_2_) leads to cytoplasmic translocation of essential transcription factors for beta-cell maturation and function, including MafA. A similar pattern of loss of transcription factors involved in maturation occurs in islets from T2D donors^4^. In line with these findings, antioxidant treatments and the overexpression of antioxidant enzymes in diabetic mouse models can delay diabetes development and/or enhances beta-cell function^9–11^.

In contrast to the damaging effect of oxidative stress in the context of insulin resistance, H_2_O_2_ plays a role in beta-cell function under normal physiological conditions. For instance, glucose-stimulated insulin secretion is impaired by the removal of H_2_O_2_^12, 13^. Additionally, treatment of pregnant mice with the antioxidant N-Acetyl cysteine (NAC) results in defects in normal beta-cell development in the offspring^14, 15^. Thus, one can hypothesize that the effect of ROS on beta-cells is context dependent. Whereas in diabetes, chronic oxidative stress could contribute to beta-cell loss and dysfunction, under physiological conditions, moderate levels of ROS maintain beta-cell function. However, whether ROS are important for additional aspects of beta-cell biology, for instance their ability to undergo expansion remains to be addressed.

In the present study we used zebrafish as an *in vivo* model to investigate the role of H_2_O_2_ in the control of beta-cell proliferation. By applying genetic and pharmacological manipulations of H_2_O_2_ levels, we show that H_2_O_2_ is required for beta-cell proliferation in response to nutrients. To support our *in vivo* findings, we also used cultured rat beta-cells as an *in vitro* model. Using a genetically-encoded reporter of H_2_O_2_, we show that glucose stimulates a rapid H_2_O_2_-increase *in vitro*, with the levels of H_2_O_2_ being important for cell expansion. Thus, our results show that H_2_O_2_ may act as a second messenger to drive beta-cell proliferation in response to changes in glucose levels.

## Results

### Endogenous H_2_O_2_ accumulation is necessary for beta-cell proliferation

**Figure 1:**
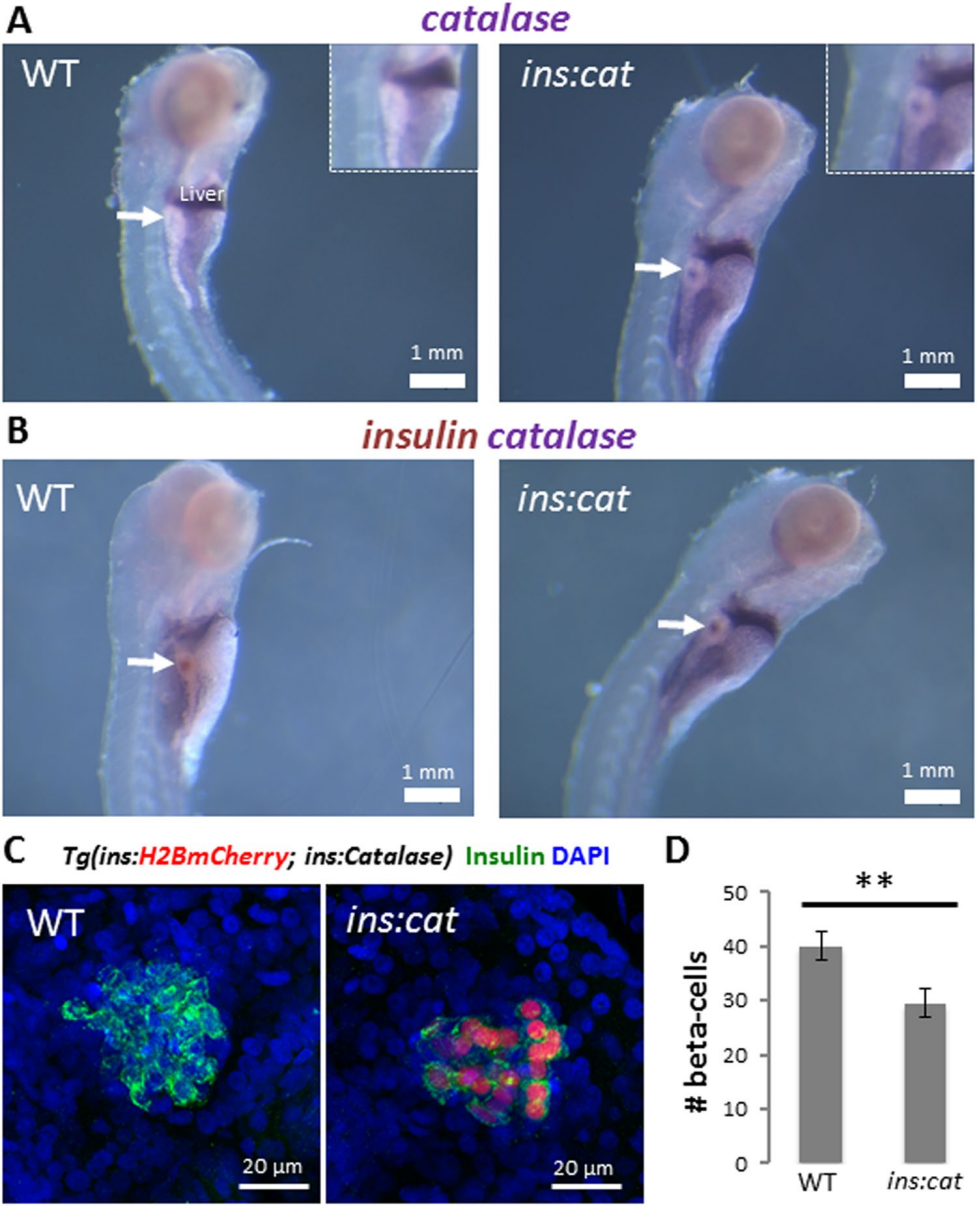
Catalase expression in beta-cells leads to a reduction in beta-cell numbers in larvae. (**A**) Whole mount *in situ* hybridization (WISH) for *catalase* in WT and *Tg*(*ins*:*catalase*;*ins*:*H2BmCherry*) larvae at 5 dpf. Arrows point to the principal islet. *Catalase* expression was not detected in the beta-cells of WT larvae whereas *Tg*(*ins*:*catalase*;*ins*:*H2BmCherry*) larvae exhibit *catalase* transcripts. (**B**) Double WISH for *insulin* (brown) and *catalase* (purple) showing overlap between the *insulin* and *catalase* transcripts in *Tg*(*ins*:*catalase*;*ins*:*H2BmCherry*) siblings. (**C**) Confocal projections of the principal islets of 4 dpf WT and *Tg*(*ins*:*catalase*;*ins*:*H2BmCherry*) larvae. (**D**) Quantification of the average number of beta-cell in WT (n = 15 larvae) and *Tg*(*ins*:*catalase*;*ins*:*H2BmCherry*) larvae (n = 12 larvae)*. Tg*(*ins*:*catalase*;*ins*:*H2BmCherry*) larvae exhibit a reduced beta-cell number compared to WT (p = 0,008, error bars = SEM).

In order to study the role of H_2_O_2_ in beta-cell proliferation, we generated transgenic zebrafish lines allowing for beta-cell specific overexpressing of the antioxidant enzyme catalase, which converts H_2_O_2_ to molecular oxygen and water. To validate that *Tg*(*ins*:*catalase*;*ins*:*H2BmCherry*) animals express *catalase* in beta-cells, we performed whole mount *in situ* hybridization for *catalase* and *insulin* at 5 dpf. While *catalase* transcripts are not detected in the beta-cells of WT larvae, *Tg*(*ins*:*catalase*;*ins*:*H2BmCherry*) larvae exhibit robust *catalase*-expression in the beta-cells (Fig. 1A and B). The absence of *catalase*-expression in WT indicates that zebrafish beta-cells express very low levels of this antioxidant enzyme.

To determine whether endogenous H_2_O_2_ production in beta-cells controls the rate of their proliferation, we examined beta-cell mass in our *Tg*(*ins*:Catalase)-expressing larvae. Interestingly, during the early larval stages (4 dpf), *Tg*(*ins*:Catalase)-expressing larvae exhibited a reduction in beta-cell number compared to WT siblings (Fig. 1C and D), suggesting that catalase overexpression inhibits beta-cell expansion. To test whether the reduction in beta-cell numbers is a result of H_2_O_2_-removal resulting from catalase overexpression in beta-cells, we incubated *Tg*(*ins*:*catalase*;*ins*:*H2BmCherry*) larvae in 200 µM H_2_O_2_ from 2 to 4 dpf. This treatment restored beta-cell numbers in the transgenic larvae, as compared to controls (Figure S1).

**Figure 2:**
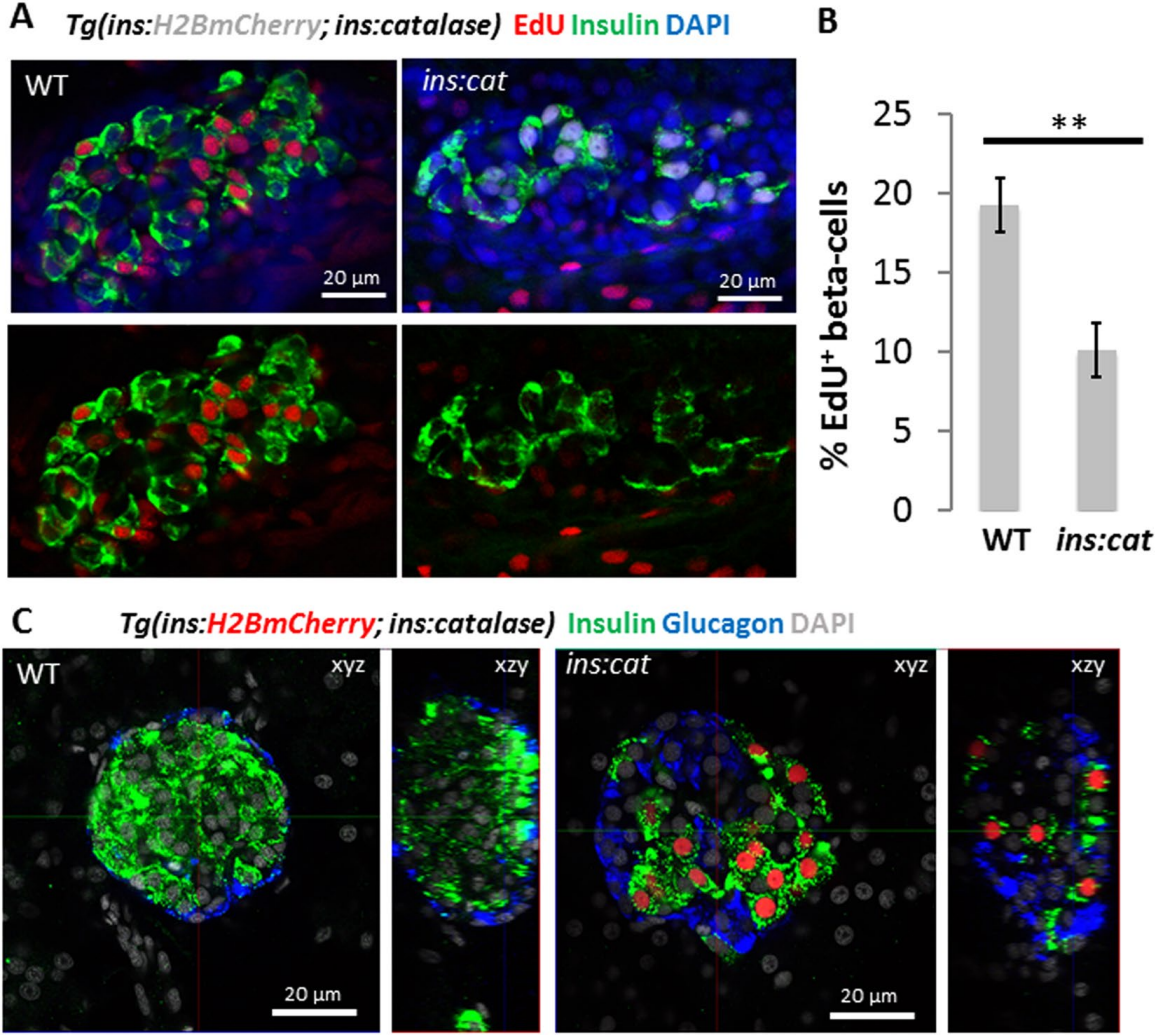
Catalase expression in beta-cells reduces beta-cell proliferation. (**A**) Confocal sections of principal islets from WT and *Tg*(*ins*:*catalase*;*ins*:*H2BmCherry*) siblings, incubated at 21 dpf with EdU for 16 hours (5 h after feeding). Fewer beta-cells incorporated EdU in *Tg*(*ins*:*catalase*;*ins*:*H2BmCherry*) compared to WT siblings. (**B**) Quantification of the percentage of EdU^+^ beta-cells in WT (n = 10) and *Tg*(*ins*:*catalase*;*ins*:*H2BmCherry*) (n = 11). Expression of catalase leads to a reduced percentage of EdU^+^ beta-cells compared to WT (p = 0,001). (**C**) Confocal planes (xzy) and 3D reconstructions (xzy) of smaller islets from WT and *Tg*(*ins*:*catalase*;*ins*:*H2BmCherry*) animals stained for insulin and glucagon. The beta-cells in the islet of the *Tg*(*ins*:*catalase*;*ins*:*H2BmCherry*) were scattered and fewer in numbers compared to WT. Alpha-cells in *Tg*(*ins*:*catalase*;*ins*:*H2BmCherry*) appear unaffected by the catalase expression in beta-cells. See also Figure S2 for maximum projections spanning the volume of principal islets in WT and *Tg*(*ins*:*catalase*;*ins*:*H2BmCherry*). Error bars = SEM.

During the late larval and juvenile stage of zebrafish development (21 to 45 dpf), high nutrient intake induces a high replication rate of beta-cells^16^. To examine whether catalase expression affects beta-cell proliferation in response to nutrients, we performed proliferation analysis using 5-ethynyl-2 deoxyuridine (EdU) uptake. WT and *Tg*(*ins*:*catalase*;*ins*:*H2BmCherry*) animals were incubated in EdU after feeding. Notably, catalase expression strongly reduced the proportion of EdU-positive beta-cells, as compared to controls (Fig. 2A and B). Consistent with this reduced proliferation rate, the islets from the *Tg*(*ins*:*catalase*;*ins*:*H2BmCherry*) animals showed reduced beta-cell mass composed of fewer and markedly scattered beta-cells compared to WT (Fig. 2C) (Figure S2). In contrast, glucagon^+^ cells appeared unaffected by the beta-cell-specific expression of catalase (Fig. 2C). We conclude that ROS accumulation in beta-cells, in particular H_2_O_2_, is necessary for their nutrient-stimulated proliferation.

### Glucose increases H_2_O_2_ levels in INS-1 cells

**Figure 3:**
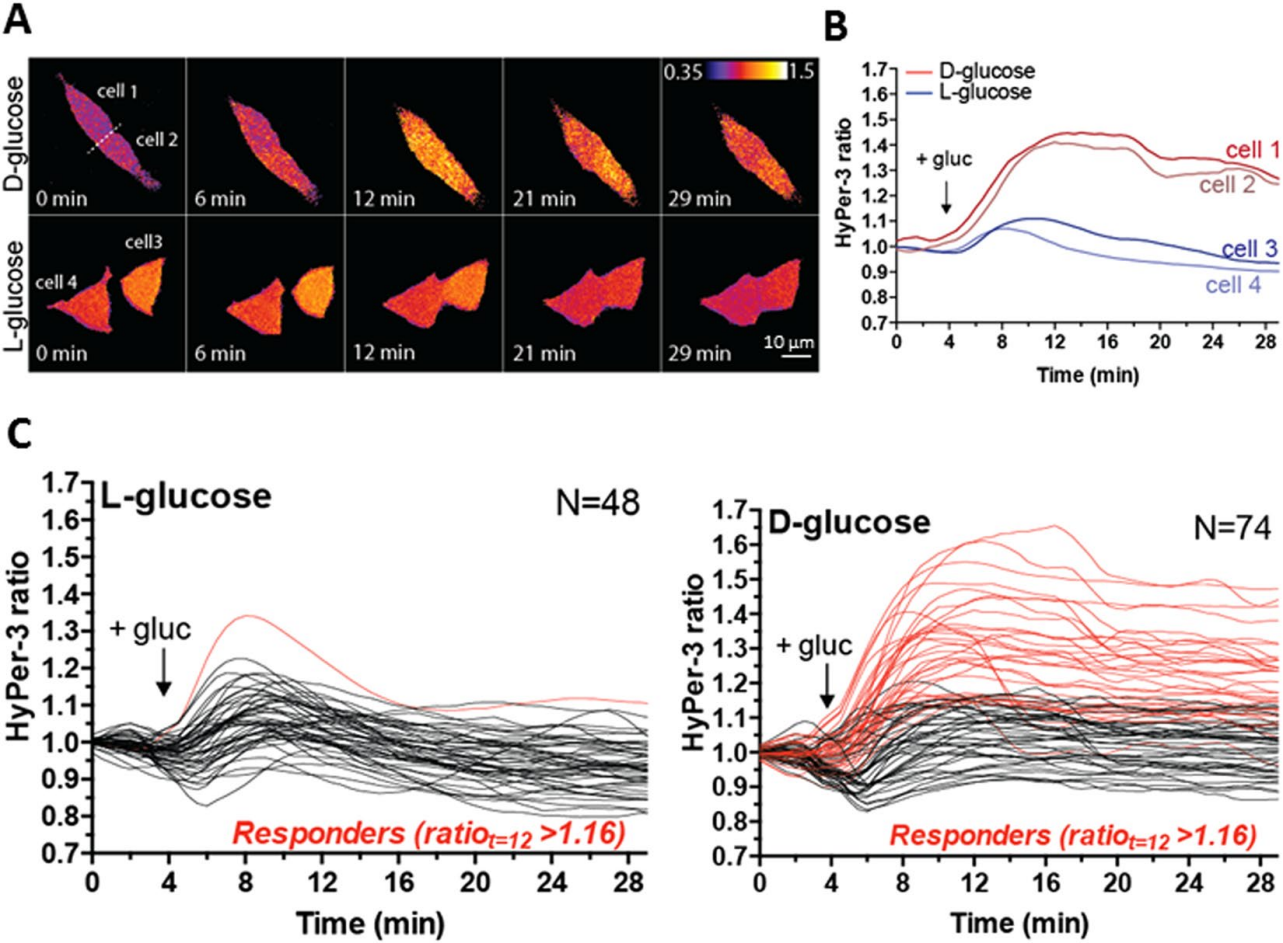
Glucose increases H_2_O_2_ levels in INS-1 cells. (**A**) Time-lapse ratio imaging of rat INS-1 cells following treatment with 18 mM D- or L-glucose at t = 4 min. (**B**) Quantification of HyPer-3 ratio of the cells shown in panel A. (**C**) Single-cell quantification of INS-1 cells treated as in panel A, showing heterogeneity in the response to D-glucose from three independent experiments. In 33 out of 74 cells, the HyPer-3 ratio rose almost immediately up to a maximum of ~1.65 in 8–10 minutes after glucose stimulation.

We hypothesized that nutrients and in particular glucose, a known mitogen for beta-cells, induce H_2_O_2_ production in the beta-cell, which then acts as a second messenger to maintain beta-cell proliferation. To test if glucose can directly increase H_2_O_2_ levels in beta-cells, we took advantage of rat INS-1 cells, which are known to respond to glucose within the physiological range^17^. To monitor glucose-stimulated changes in H_2_O_2_ levels, INS-1 cells were transfected with HyPer-3, and stimulated with 18 mM D-glucose (Fig. 3A and B). HyPer-3 allows for ratiometric measurements of H_2_O_2_ levels, as it exhibits two excitation peaks at 420 nm and 488 nm, and one emission peak at 516 nm that depend on the intracellular levels of H_2_O_2_. Upon an increase in H_2_O_2_ levels, the 420 nm excitation peak decreases proportionally to an increase in the 488 nm excitation peak^18^. In 33 out of 74 cells, the HyPer-3 ratio rose almost immediately up to a maximum of ~1.65 about 8–10 minutes after glucose stimulation (Fig. 3C). Despite all cells experiencing the same glucose stimulus, we noticed that the dynamics of the H_2_O_2_ increase differed among individual cells. Some cells showed shorter bursts of H_2_O_2_ whereas in others the levels of H_2_O_2_ remained elevated during the course of the experiment (Figure S3). The remaining cells did not, or only weakly responded to D-glucose (maximum HyPer-3 ratio < 1.16, see material and methods) similar to control cells that were stimulated with L-glucose (Fig. 3C). This heterogeneity in responses could be due to the pre-existing differences in the metabolic or proliferative states of INS-1 cells at the time of glucose-treatment. Together these data indicate that direct glucose stimulation increases H_2_O_2_ levels.

### Different levels of H_2_O_2_ control beta-cell proliferation and quiescence *in vivo* and *in vitro*

**Figure 4:**
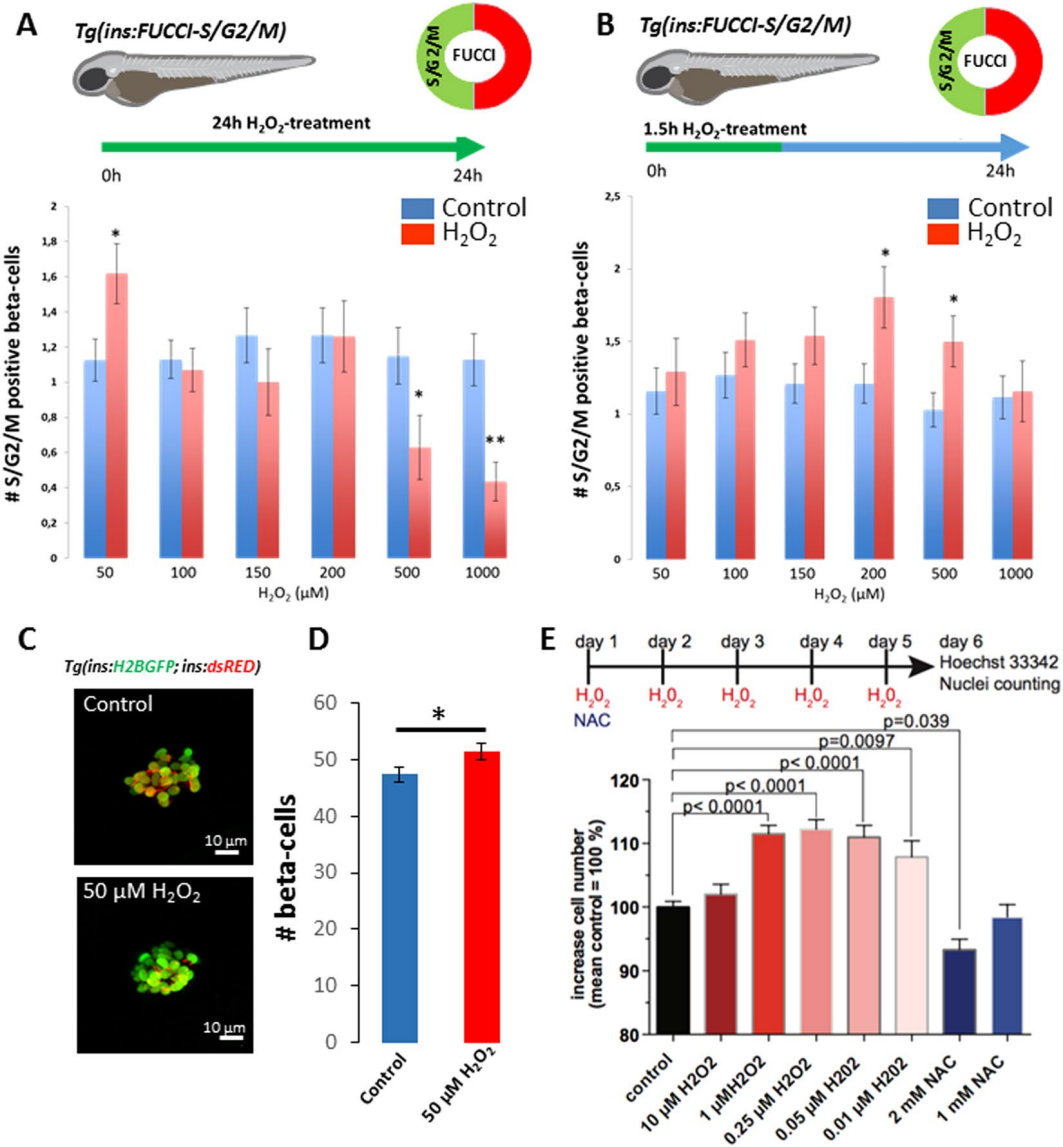
Different levels of H_2_O_2_ induce beta-cell proliferation and quiescence. (**A**) Quantification of the number of *Tg*(*ins*:FUCCI-S/G2/M)^+^ beta-cells after continuous treatment with increasing concentrations of H_2_O_2_ from 3 to 4 dpf. The treatment with 50 μM H_2_O_2_ (n = 89 larvae) increased the number of *Tg*(*ins*:FUCCI-S/G2/M)^+^ beta-cells compared to controls (n = 104 larvae) (p = 0.025) whereas the treatments with 500 and 1000 μM H_2_O_2_ decreased the number of *Tg*(*ins*:FUCCI-S/G2/M)^+^ beta-cells (n = 38 larvae for 500 H_2_O_2_ μM; n = 63 larvae for controls; p = 0.03) (n = 46 larvae for 1000 μM H_2_O_2_; n = 78 larvae for controls; p = 0.0004). (**B**) Quantification of the number of *Tg*(*ins*:FUCCI-S/G2/M)^+^ beta-cells in 3 dpf larvae treated with increasing concentrations of H_2_O_2_ for 1.5 hours followed by a wash-out. The number of *Tg*(*ins*:FUCCI-S/G2/M)^+^ beta-cells was examined 24 hours after the initiation of the treatment. The short treatment with 200 and 500 μM H_2_O_2_ increased the number of *Tg*(*ins*:FUCCI-S/G2/M)^+^ beta-cells compared to controls (n = 51 larvae for 200 μM H_2_O_2_; n = 86 larvae for controls; p = 0.02) (n = 68 larvae for 500 μM H_2_O_2_; n = 96 for larvae for controls; p = 0.02). (**C**) Confocal projections of the principal islets of 4 dpf *Tg*(*ins*:*H2B-GFP; ins*:*dsRED*) larvae treated from 3 to 4 dpf with 50 μM H_2_O_2_. (**D**) Quantification of the average number of beta-cells marked with H2B-GFP in controls and H_2_O_2_-treated larvae. H_2_O_2_-treatement increased beta-cell number (n = 30 larvae) compared to controls (n = 26 larvae) (p = 0,046). Error bars = SEM. (**E**) Bar chart showing the increase in the number of INS-1 cells after H_2_O_2_ or NAC-treatments. Data from four independent experiments and > 3.5 × 10^5^ cells per condition were normalized to the mean of control treated cells ( = 100%) and analyzed for significance using a one-way ANOVA with Dunnett’s multiple comparison test. Error bars indicate S.E.M. Cartoons with permission by Luis Delgadillo.

To investigate if an increase in H_2_O_2_ levels is sufficient to promote beta-cell proliferation, we modulated the levels of H_2_O_2_ by incubating 3 dpf zebrafish larvae in increasing concentrations of H_2_O_2_ (from 50 μM to 1 mM) (Fig. 4A) and over different durations (Fig. 4B) while scoring beta-cell proliferation using our beta-cell specific fluorescence ubiquitination cell cycle indicator (FUCCI)-S/G2/M-reporter line^19^. In this system, beta-cells that enter S-phase and progress through the cell cycle exhibit green-fluorescence, allowing to quantify beta-cell replication non-invasively. Strikingly, we found that treatment with lower concentrations of H_2_O_2_ (50 μM) for 24 h, increased cell-cycle entry compared to controls, whereas treatment with high concentrations of H_2_O_2_ (1 mM) decreased it (Fig. 4A). Furthermore, a short 1.5 h-treatment with intermediate concentrations of H_2_O_2_ (200 and 500 μM) followed by a wash-out also led to an increase in cell-cycle entry 24 h later (Fig. 4B). These data indicate that a prolonged but moderate increase in H_2_O_2_ levels (50 μM H_2_O_2_ for 24 h) as well as a transient burst of H_2_O_2_ (200–500 μM H_2_O_2_ for 1.5 h) are sufficient to trigger cell-cycle entry in beta-cells. In contrast, the prolonged treatment with high concentrations of H_2_O_2_ is inhibitory to beta-cell proliferation. In line with our findings using the *ins*:FUCCI-S/G2/M-reporter, treatment with 50 μM H_2_O_2_ caused an increase in total beta-cell numbers compared to controls (Fig. 4C and D), indicating that low levels of H_2_O_2_ cause a productive entry in the cell cycle. Finally, we determined if H_2_O_2_-treatment causes beta-cell toxicity and cell death. We performed terminal deoxynucleotidyl transferase dUTP nick end labeling (TUNEL) in the pancreas of larvae treated with 50 µM and 1 mM H_2_O_2_ from 3 to 4 dpf. We did not observe TUNEL-positivity in the beta-cells or in the surrounding pancreatic tissue from the control or the H_2_O_2_-treatment groups (Figure S4A–C). TUNEL^+^ beta-cells were only observed in the positive controls, in which we experimentally induced targeted beta-cell ablation (Figure S4D).

It must be noted that the concentration range (50 µM to 1 mM H_2_O_2_) that we used *in vivo* might seem un-physiological if directly applied to cells, however, the actual concentration that reaches the internal organs of the zebrafish larvae *in vivo* is likely much lower^20, 21^. To directly test if lower levels of H_2_O_2_ levels can promote proliferation when applied directly to mammalian beta-cells, we treated INS-1 cells for five days with low, nontoxic levels of exogenously added H_2_O_2_ or with the reducing agent N-acetyl-cysteine (NAC) as a negative control. Because H_2_O_2_ is metabolized within an hour in cell culture medium^22^, we supplied H_2_O_2_ every 24 hours, while NAC was only added in the beginning of the experiment. Treatment of INS-1 cells with 0.25 μM H_2_O_2_ produced a significant increase in cell numbers compared to controls (Fig. 4E). Only addition of low levels of H_2_O_2_, ranging from 0.01 to 1 μM, promoted cell proliferation in a dose-dependent manner, whereas higher levels of H_2_O_2_ (10 μM) did not show a stimulatory effect. In line with the positive effect of H_2_O_2_ on proliferation, reducing ROS levels with 2 mM NAC decreased proliferation (Fig. 4E). These results show a conserved role of H_2_O_2_ in stimulating beta-cell proliferation with much lower concentrations eliciting a proliferative response *in vitro* as compared to *in vivo*.

### High levels of H_2_O_2_ promote beta-cell neogenesis

**Figure 5:**
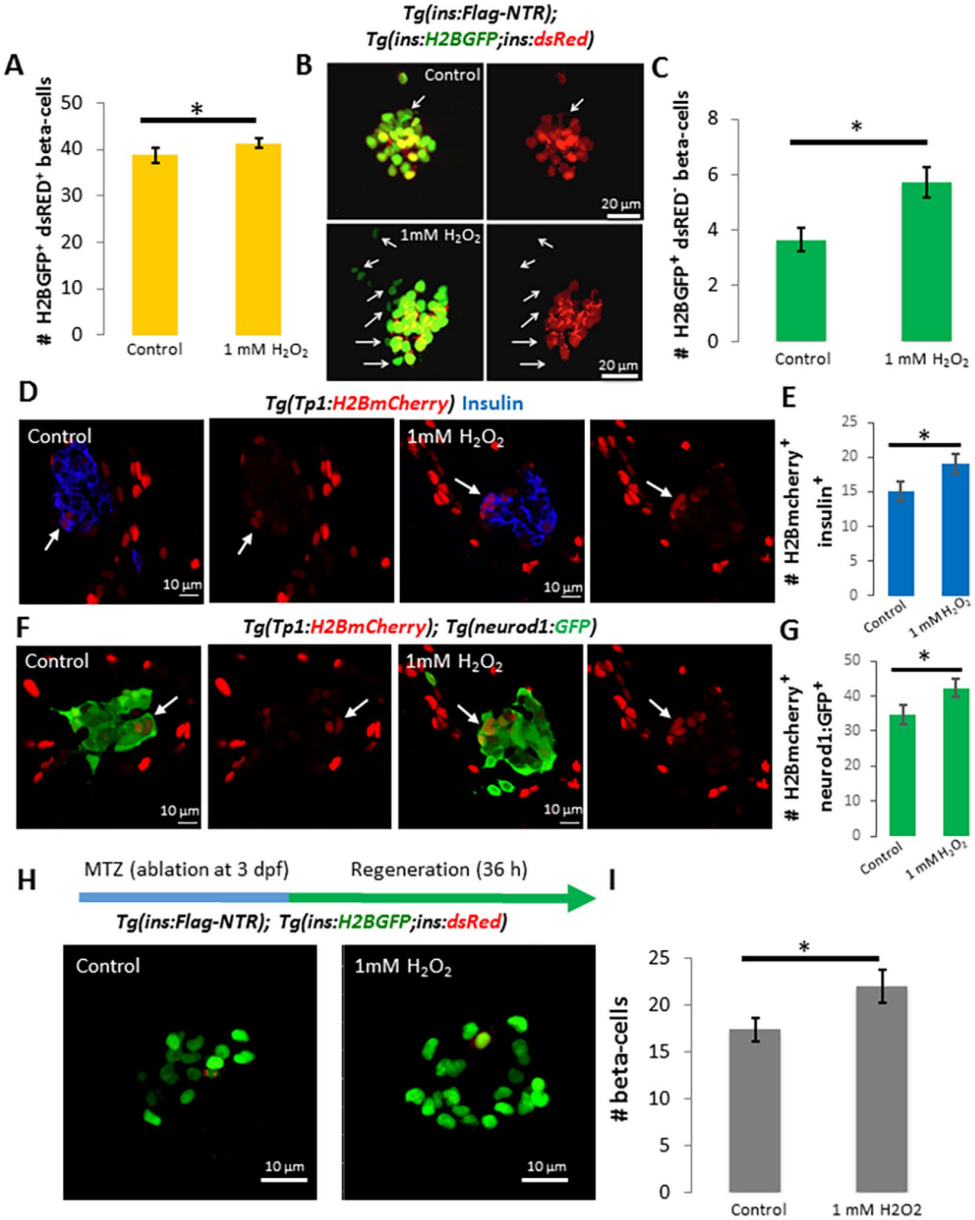
High levels of H_2_O_2_ promote new beta-cell neogenesis and regeneration. (**A**) Quantification of the average number of beta-cells marked with H2B-GFP in controls (n = 22) and larvae treated with 1 mM H_2_O_2_ from 3 to 4 dpf (n = 23). The larvae treated with 1 mM H_2_O_2_ exhibit an increase in the number of beta-cells compared to controls (p = 0,04). (**B**) Confocal projections of the principal islet of 4 dpf WT and *Tg*(*ins*:*H2B-GFP*; *ins*:*dsRED*) larvae treated with vehicle or 1 mM H_2_O_2_ from 3 to 4 dpf. Arrowheads point to H2B-GFP^+^ dsRED^−^ cells. (**C**) Quantification of the number of H2B-GFP^+^ and dsRED^−^ cells in controls and H_2_O_2_-treated larvae. Beta-cells that are H2B-GFP^+^ but dsRED^−^ represent recently-formed cells due to the slower maturation of dsRED compared to GFP. H_2_O_2_-treatment increased the number H2B-GFP^+^ and dsRED^−^ beta-cells compared to controls (p = 0,004), indicating an increase in new beta-cell formation. Error bars = SEM. (**D**) Confocal sections of primary islets from *Tg*(*Tp1*:*H2BmCherry*) larvae stained for insulin (blue). *Tg*(*Tp1*:*H2BmCherry*) drives expression of a fluorescent protein with long half-life (H2BmCherry) in the Notch responsive cells (NRCs) in the pancreas. Beta-cells that differentiate from NRCs can retain H2BmCherry-flurescence due to perdurance. The larvae were treated with vehicle or 1 mM H_2_O_2_ from 3 to 4 dpf. The arrows indicate *Tp1*:H2BmCherry^+^ and insulin^+^ cells in the periphery of the primary islets in controls and H_2_O_2_-treated larvae. See Figure S6 for higher resolution images. (**E**) Quantification of the average number of *Tp1*:H2BmCherry^+^ and insulin^+^ cells in the principal islets of controls (n = 21) and H_2_O_2_-treated larvae (n = 19), showing an increase following the H_2_O_2_-treatement (p = 0,049). (**F**) Confocal sections of primary islets from *Tg*(*Tp1*:*H2BmCherry*); *Tg*(*neurod1-GFP*) larvae. The arrows indicate *Tp1*:H2BmCherry^+^ and GFP^+^ cells in the periphery of the primary islets in controls and H_2_O_2_-treated larvae. See Figure S7 for higher resolution images. (**G**) Quantification of the average number of *Tp1*:H2BmCherry^+^ and GFP^+^ cells in the principal islets of controls (n = 21) and H_2_O_2_-treated larvae (n = 19), showing an increase following the H_2_O_2_-treatement (p = 0,046). (**H**) Confocal projections of *Tg*(*ins*:*FLAG-NTR*); *Tg*(*ins*:*H2B-GFP*;*ins*:*dsRED*) larvae. Beta-cells were ablated by incubating larvae in MTZ at 3 dpf. Subsequently, the larvae were treated with vehicle or 1 mM H_2_O_2_. (**I**) Quantification of the average number of beta-cells in controls (n = 24) and H_2_O_2_-treated larvae (n = 22). H_2_O_2_-treatment significantly increased the number of regenerating beta-cells (p = 0,03). Note that a majority of the regenerated beta-cells are H2B-GFP^+^ and dsRED^−^. Error bars = SEM.

Although treatment with 1 mM H_2_O_2_ for 24 h inhibited beta-cell proliferation in zebrafish, we were intrigued to find that the total number of beta-cells in H_2_O_2_-treated larvae exhibited a slight and significant increase compared to controls (Fig. 5A). This data suggests that while high concentrations of H_2_O_2_ block beta-cell proliferation, they might increase beta-cell numbers via other means, such as the formation of new beta-cells by neogenesis. To test this option, we utilized a double transgenic system that allows distinguishing newly differentiated from older beta-cells. In this system, the *insulin* promoter drives simultaneous expression of H2B GFP and dsRED. Due to the significantly faster maturation of GFP compared to dsRED, those beta-cells that exhibit GFP but no dsRED expression are newly-formed while older beta-cells co-express both GFP and dsRED^16^. Thereby, we incubated larvae with a high dose of H_2_O_2_ (3 to 4 dpf) and then we counted the number of H2B GFP-single^+^ cells in the principal islet. As anticipated, H_2_O_2_-treated larvae exhibited an increase in the number of newly-formed beta-cells, as compared to controls (Fig. 5B and C). Furthermore, using *Tg*(*mnx1*:*GFP*), which labels immature beta-cells on their way to differentiation^23, 24^, we found a significant increase in the number of *Tg*(*mnx1*:GFP)^+^ and insulin-negative cells in the periphery of the principal islet after treating larvae with H_2_O_2_ (Figure S5). In conclusion, although 1 mM H_2_O_2_ inhibits proliferation of the beta-cells, it leads to an increase in the beta-cell mass through stimulating beta-cell neogenesis.

To identify the progenitors that could account for an increase in beta-cell differentiation in response to increasing levels of H_2_O_2_, we studied the role of the pancreatic Notch responsive cells (NRCs), which give rise to new beta-cells during development^25, 26^. To follow NRC differentiation into beta-cells, we examined *Tg*(*Tp1*:*H2BmCherry*), a transgenic line that drives the expression of a fusion protein between Histone2B (H2B) and mCherry in NRCs. Given that H2BmCherry has a long half-life, beta-cells that differentiate from the NRCs retain H2BmCherry-flurescence due to perdurance^16^. To test if increasing the levels of H_2_O_2_ would increase the number of beta-cells that differentiate from NRCs, we treated larvae with 1 mM H_2_O_2_ from 3 to 4 dpf and quantified the number of *Tp1*:H2BmCherry^+^ and insulin^+^ cells in the primary islet. While *Tp1*:H2BmCherry^+^ and insulin^+^ cells were observed in both controls and H_2_O_2_-treated larvae, their numbers were higher following the treatment with H_2_O_2_ (Fig. 5D and E) (Figure S6). In addition, we analyzed the expression of *neurod1*, which marks the commitment to the endocrine-lineage^27^. *Tg*(*neurod1*:*GFP*); *Tg*(*Tp1*:*H2BmCherry*) larvae were treated with 1 mM H_2_O_2_ from 3 to 4 dpf. Consistent with the results from the beta-cell-tracing experiment, the H_2_O_2_-treated larvae exhibited an increase in the numbers of *Tp1*:H2BmCherry^+^ and GFP^+^ cells in the primary islet (Fig. 5F and G) (Figure S7), suggesting that higher levels of H_2_O_2_ increase the differentiation of NRCs towards the endocrine lineages.

We tested if the H_2_O_2_-mediated induction of beta-cell neogenesis can enhance beta-cell regeneration after near-complete ablation of the beta-cells. For temporal ablation of beta-cells, we used a *Tg*(*ins*:*NTR*) line, which expresses the Nitroreductase (NTR) enzyme from *Escherichia coli* under the *insulin* promoter. The NTR enzyme converts the pro-drug metronidazole (MTZ) to its cytotoxic form, leading to the specific ablation of beta-cells^28^. We ablated the beta-cells at 3 dpf, followed by incubation with H_2_O_2_ during regeneration. Using the *Tg*(*ins*:*H2B-GFP*; *ins*:*dsRED*) line, we observed that a majority of the regenerating beta-cells in both controls and H_2_O_2_-treated larvae were H2B-GFP^+^ but dsRED^−^ (Fig. 5H), indicating that these regenerating beta-cells arise by neogenesis rather than replication of pre-existing beta-cells that escape ablation. Notably, the H_2_O_2_-treatement increased the numbers of beta-cells post-ablation, indicating that higher levels of H_2_O_2_ can enhance regeneration (Fig. 5H and I).

## Discussion

**Figure 6:**
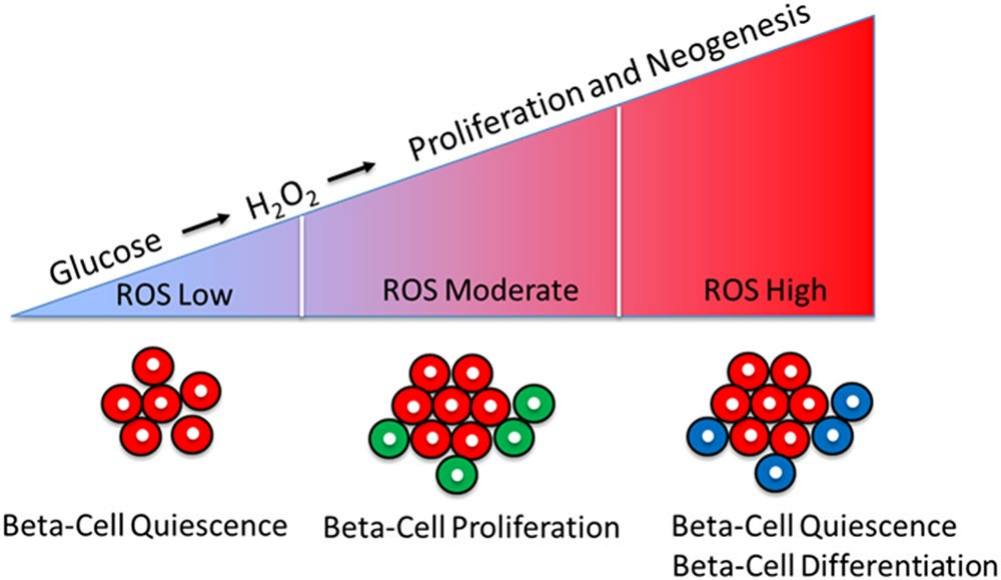
Model showing the role of different levels of H_2_O_2_ in beta-cell proliferation and neogenesis. A regular supply of low levels of exogenous H_2_O_2_ is necessary for beta-cell proliferation. Moderate levels of H_2_O_2_ drive beta-cell proliferation, whereas high levels of H_2_O_2_ induce beta-cell quiescence and stimulate neogenesis of beta-cells. Our model suggests that in a physiological context, such as nutrient-stimulation, H_2_O_2_ is produced by the beta-cell in response to glucose and plays a role as a second-messenger responsible for driving beta-cell proliferation.

Our study provides evidence that a regular supply of low levels of exogenous H_2_O_2_ is necessary for beta-cell proliferation. Moderate levels of H_2_O_2_ promote beta-cell proliferation, whereas high levels of H_2_O_2_ levels induce beta-cell quiescence. Interestingly, while high levels of H_2_O_2_ inhibit proliferation, they can stimulate differentiation of new beta-cells from progenitors (Fig. 6). Our model also suggests that in a physiological context such as nutrient intake, H_2_O_2_ is produced by the beta-cell in response to glucose, and plays a role as a second-messenger in promoting beta-cell proliferation, thereby coordinate metabolic activity with beta-cell mass plasticity. These findings highlight that a fine balance of H_2_O_2_ levels should be maintained within the beta-cells to ensure proper function and ability for undergo metabolic compensation. Notably, during the final revision of our paper, the group of Maike Sander published a new study using a genetic mouse model allowing to overexpress catalase specifically in beta-cell mitochondria. The overexpression of catalase caused significant reduction in the percentage of proliferating beta-cells and a reduction in total beta-cell mass compared to controls^29^. Altogether, these results point to an evolutionary conserved role of H_2_O_2_ -production in maintaining beta-cell proliferation.

In addition to our work, recent studies have implicated the levels of ROS as important regulators of cell cycle progression and renewal in stem cells, including spermatogonial^30^, basal airway^31^, and neural stem cells^32^, pointing towards a broad mechanism of oxidative-control of cell cycle progression. Interestingly, in neuronal stem cells, ROS regulate the activation of the PI3K-Akt pathway^32^. Furthermore, Wnt signaling pathway is regulated by H_2_O_2_^33^. Interestingly, both Wnt and PI3-Akt pathways are involved in positively regulating beta-cell proliferation^34–36^. Whether PI3-Akt and/or Wnt signaling pathways are modulated by H_2_O_2_ in our model to drive beta-cell proliferation remains to be elucidated.

Previous work in rodents and pancreatic explants illustrated a role for H_2_O_2_ in beta-cell differentiation during development and regeneration^14, 15^. Similarly, we show that treatment with high concentrations of H_2_O_2_ stimulates beta-cell neogenesis and promotes regeneration in zebrafish. The similarity between all these findings highlights a conserved and important role of H_2_O_2_ in the control of beta-cell neogenesis. However, if H_2_O_2_ regulates differentiation directly by acting on the progenitors, or if it acts by inducing modifications in the progenitor niche that favor differentiation, still remains to be investigated. For example, previous work in zebrafish suggested that prolonged activation of insulin secretion from beta-cells can induce compensatory beta-cell differentiation^37^. Interestingly, we observed a decrease in glucose levels in larvae treated with high concentrations of H_2_O_2_ (data not shown), which may indicate activation of the beta-cells that in turn promote new beta-cell differentiation via insulin secretion. It will be important to develop new tools allowing for cell type-specific manipulations of H_2_O_2_ levels in order to address better the role of ROS in progenitor differentiation.

Finally, previous studies have shown the beneficial role of antioxidant-treatment or overexpression of antioxidant enzymes in protection against diabetes^9–11^. Moreover, the transcription factor Nrf2, which is responsible for the regulation of the expression of antioxidant enzymes can protect beta-cells against reactive nitrogen species (RNS), and prevent diabetes development in db/db mice^38, 39^. In contrast, we provide data showing the under physiological conditions, the beta-cell-specific reduction of ROS levels via catalase expression leads to negative effects, as it impairs beta-cell mass expansion. Hence, the proposed beneficial role of antioxidant therapy in diabetes should be revisited, as in the longer term, it might compromise the beta-cells, since ROS clearly play a physiological role in these cells.

## Materials and Methods

### Zebrafish lines

Wild-type or transgenic zebrafish of the outbred AB, WIK or a hybrid WIK/AB strain were used in all experiments. Zebrafish were raised under standard conditions at 28 °C. Published transgenic strains used in this study were:

*Tg*(*ins*:*mAGFP-gmnn,cryaa*:*mCherry*)^*947*^ ^19^; *Tg*(*ins*:*FLAG-NTR,cryaa*:*mCherry*)^*s950*^ ^40^; *Tg*(EPV. Tp1-Mmu. Hbb:*hist2h2l-mCherry*)^*s939*^ ^26^; *TgBAC*(*neurod1*:*EGFP*)^41^; *Tg*(*ins*:*CFP-NTR*)^*s892*^ ^28^; *Tg*(*ins*;*Hsa.HIST1H2BJ-GFP*;*ins*:*DsRed*)^*s960*^ ^16^ and *Tg*(*mnx1*:*GFP*)^42^. Experiments were conducted in accordance with the Animal Welfare Act and with permissions approved by Landesdirektion Sachsen (AZ 24–9168.11-1/2013-14, TV38/2015, T12/2016, T13/2016, Germany).

### Generation of *Tg*(*ins*:*catalase*;*ins*:*H2BmCherry*)

The cDNA encoding the zebrafish *catalase* gene was PCR-amplified and cloned downstream of the *insulin* promoter. The vector also contains a second *insulin* promoter driving expression of H2BmCherry as well as YFP, which is expressed under the *cryaa* promoter for identification of transgenic animals by retinal-expression of YFP. Transgenics were generated using the tol2-system.

### Generation of probes for *in situ* hybridization

*Catalase* cDNA was PCR amplified using the forward primer 5′-tttt-GAATTC-atggcagacgacagagaaaag-3′ and the reverse primer 5′-ttttTCTAGAtcacatcttagaagctgcagC-3′. The PCR product was sub cloned in PCS2 vector. For antisense probe synthesis, the vector was linearized by digestion with BamHI. DIG RNA labelling mix (Roche) was used to generate DIG-labelled *catalase* probes by *in vitro* transcription with the T7 RNA polymerase (Fermentas). RNA probes were purified using the Micro Bio-Spin 30 Columns, Tris, RNase-free (Bio-Rad). cDNA from 24 hpf embryos was PCR amplified using the appropriate primers for the insulin gene *insa*- fw: 5′atg gca gtg tgg ctt cag gc3′insa-rv: 5′gaa ttc tca gtt aca gta gt 3′. PCR products of the correct size were purified from agarose gels using the QIAquick® Gel Extraction Kit (QIAGEN), and subcloned using the Dual Promoter TA Cloning® kit (Invitrogen). DNA was sequenced to confirm that inserts correspond to the correct gene and to determine orientation of the insert. Approximately 15 μg of plasmid was digested with 5 U of SpeI enzyme over night at 37 °C followed by heat inactivation of the enzyme for 10 min at 65 °C. The digested plasmid was then gel-purified. For the *in vitro* transcription reaction, approximately 3 μg of linearized DNA, 2 μl of fluorescein labeled DNTP’s (Roche), 4 μl 5× transcription buffer (Fermentas), 1 μl RibolockTM RNase Inhibitor (Fermentas) and 2 μl of T7 RNA polymerase (Fermentas) were mixed and filled up to 20 μl with DEPCddH2O. The reaction mixture was incubated at 37 °C for 3 hours followed by adding 2 μl DNaseI (Fermentas) and incubation for another 20 min at 37 °C to digest the template DNA. The transcription reaction was terminated by adding 50 mM EDTA(Fermentas). RNA probes were then cleaned up using the Micro Bio-Spin 30 Columns, Tris, RNase-free (Bio-Rad) and filled up with 100 μl Hyb + for storage at −20 °C.

### Whole mount *in situ* hybridization

5 dpf WT and *Tg*(*ins*:*catalase*;*ins*:*H2BmCherry*) siblings were fixed in fresh 4% PFA then dehydrated using a methanol gradient. Samples were rehydrated using the reverse order of the methanol gradient and washed three times in 1X DEPC-PBST. Samples were then incubated in proteinase K, which was washed out by 1X DEPC-PBST. Samples were re-fixed in 4% PFA for 20 minutes at RT. PFA was washed out by 1xDEPC-PBST. Samples were incubated in hybridization (Hybe) buffer for 2 hours at 65 °C. Shortly before applying the probes (1 ng/µl), each probe was denatured in Hybe for 10 minutes at 75 °C. Samples were incubated with the DIG-labeled *catalase* probe and the fluorescein-labeled *insulin* probe in Hybe solution O.N. at 65 °C. Samples were then washed at 65 °C in: [3xSSC/50% formamide/0.1% Tween-20]; [2xSSC/50% formamide/0.1% Tween-20]; [2xSSC/0.1% Tween-20; 0.2xSSC/0.1% Tween-20], followed by washes with 1xPBST and subsequent washes with maleic buffer at RT. Afterwards, samples were incubated in 2% blocking buffer (Roche) for 2 hours at RT. To detect the DIG-labeled RNA probes, samples were incubated in anti-DIG-AP-fab fragments (Roche) diluted 1:5000 in blocking buffer over night at 4 °C. Samples were rinsed with maleic buffer, 1xPBST and NTMT. For staining the substrates, NBT and BCIP were added to the NTMT buffer. Signal development was monitored every 15 minutes under a stereomicroscope and stopped with 1xPBST washes. To detect the *insulin* signal, samples were incubated in anti-Fluorescein AP 1:2000 in blocking buffer O/N at 4 degrees. For fluorescein signal detection, samples were incubated in NTMT staining buffer with the substrates INT and BCIP. Signal development was monitored under a stereomicroscope and stopped with washes in 1xPBST.

### Immunostaining and imaging

Protocol for whole mount immunostaining was adapted from^26^. Primary antibodies used were anti-Insulin (Guinea Pig from DAKO 1:200), anti-GFP (Chicken from Abcam 1:1000) and anti-Glucagon (Mouse from Sigma 1:400). Secondary antibodies used were Alexa Fluor antibodies (1:300). Z-Stacks were obtained using a LSM-780 confocal microscope unless otherwise indicated in the text. For imaging of *Tg*(*ins*:*Hsa.HIST1H2BJ-GFP*;*ins*.*dsRED*)^*s960*^, images were acquired by setting the gain just below the threshold of signal saturation in both GFP and dsRed channels. For image analysis, the number of green only beta-cells was counted using ZEN blue software. Beta-cells which did not have an overlapping GFP and dsRED signals were counted as GFP-positive but dsRED-negative beta-cells.

### TUNEL assay

To detect beta-cell death we used the Click-iT® TUNEL Alexa Fluor 647 kit (Invitrogen: C10247). Larvae were fixed in 4% PFA in PBS for 48 hours at 4 degrees under constant mixing. The larvae were washed in 1XPBS and dissected to remove the skin covering the islet. Dissected larvae were permeabilized by incubation in 1% Triton X-100 in 1X PBS (1%PBST) for 20 minutes (3 times). Next, we prepared the TdT reaction according to kit protocol. 200 µl of TdT reaction mixture were used per 6 larvae. Larvae were incubated in the TdT reaction at 37 °C for one hour. The larvae were washed 2 times each for 2 minutes in 4% BSA in 1%PBST. The Click it reaction was prepared according to Kit protocol and 200 µl of reaction mixture were used per 6 larvae. Larvae were incubated at room temperature and protected from light for 30 minutes. The larvae were washed with 4% BSA in 1%PBST for 5 minutes. Finally, to detect the *ins*:CFP signal, which is lost following the Click-iT®reaction, we performed antibody staining using anti-GFP antibody as described in the immunostaining and imaging section.

### Drug and H_2_O_2_ treatment

Hydrogen peroxide (H1009, SIGMA) was diluted in E2 or E3 medium to the final concentrations indicated in the text. Treatments were performed as indicated in the text. For beta-cell ablation, larvae were incubated with 10 mM MTZ (Sigma, M1547) dissolved in aquarium water, as described previously^28^. Following ablation, MTZ was washed away by rinsing the larvae and transferring them to fresh medium.

### Analysis of proliferation using EdU

Equal numbers of 21 dpf WT and *Tg*(*ins*:*catalase*;*ins*:*H2BmCherry*) siblings were fed with brine shrimp (Artemia). 5 h after feeding, the animals were placed in fish water with 2.5 mM EdU for 16 hours. The animals were euthanized and dissected, EdU detection was performed according to the kit protocol Click-iT® EdU Alexa Fluor® 647 Imaging Kit (C10340 Thermo).

### Culture, stimulation and imaging of INS-1 cells

Rat insulinoma INS-1 cells (a gift from Prof. M. Solimena, PLID, Dresden, Germany) were cultured in RPMI 1640 medium (Gibco) supplemented with 20 mM HEPES, 10% heat‐inactivated fetal calf serum, 2 mM L‐glutamine, 100 U/ml penicillin, 100 μg/ml streptomycin, 1 mM sodium pyruvate and 50 μM 2‐mercaptoethanol as described previously^17^. For transient transfection, INS-1 cells were electroporated using a Neon Transfection system (Thermofisher) according to the manufactures instructions. 3 days post transfection 15 × 10^3^ cells were seeded in a 96 well plate. The following day cells were washed, pre-incubated for 1 h in resting buffer (5 mM KCl, 120 mM NaCl, 24 mM NaHCO_3_, 1 mM MgCl_2_, 2 mM CaCl_2_, 1 mg/ml bovine serum albumin, 15 mM HEPES pH 7.4) and imaged on an ImageXpress Micro XLS (Molecular Devices) using a 40x air objective. After 4 minutes cell were stimulated by either 18 mM D-glucose/40 mM KCl or 18 mM L-glucose/40 mM KCl and imaged for 25 min more. Single cells were imaged on a MetaXpress Micro XLS (Molecular Devices) and excited using 500/24 and 438/24 filters using a Spectra X light engine (Lumencor). Ratiometric image analysis was performed according to^43^ using Fiji. Values were normalized to the ratio at the beginning of the experiment and plotted over time after smoothing (4 neighbors, 6^th^ order polynomial). The response threshold (responders) was 1.16 reflecting the mean + 2 SD of L-glucose treated cells at t = 12 min.

### INS-1 treatment with H_2_O_2_ and N-acetyl-cysteine

22 × 10^3^ cells were seeded in 96 well plates the day before the experiment. Cells were either treated once with 1-2 mM N-acetyl cysteine (Sigma) at the beginning of the experiment or every 24 hours with the indicated concentrations of H_2_O_2_. After five days 10 μg/ml Hoechst 33342 was added to the cells and the total wells were imaged using a 10x air objective on an ImageXpress Micro XLS (Molecular Devices). The nuclei of all cells were segmented and counted using the MetaXpress (Molecular Devices) and data were normalized to the mean of control-treated cells.

### Statistical analysis

All results are presented as mean ± standard error of the mean (SEM) unless otherwise indicated. Data were analyzed using Student’s t-test or ANOVA. A P-value of less than 0.05 was considered statistically significant.

## Electronic supplementary material

Supplementary information
