# Supplementary material for: Distinct Levels of Reactive Oxygen Species Coordinate Metabolic Activity with Beta-cell Mass Plasticity
Source: Sci Rep. 2017 Jun 26;7:3994. doi: 10.1038/s41598-017-03873-9 (PMC5484671; doi:10.1038/s41598-017-03873-9)
Supplement: Supplementary file 1 — Supplementary information [file 41598_2017_3873_MOESM1_ESM.pdf]

Supplementary information

Distinct Levels of Reactive Oxygen Species Coordinate Metabolic Activity with Beta-cell Mass Plasticity

Ezzaldin Ahmed Alfar[1,2,3] *, Dilyana Kirova[4] *, Judith Konantz[1,2], Sarah Birke[1,2,], Jörg Mansfeld[4]** and Nikolay Ninov[1,2,3]**

* These authors contributed equally

** Correspondence to joerg.mansfeld@biotec.tu-dresden.de and nikolay.ninov@crt-dresden.de

Supplementary Figures

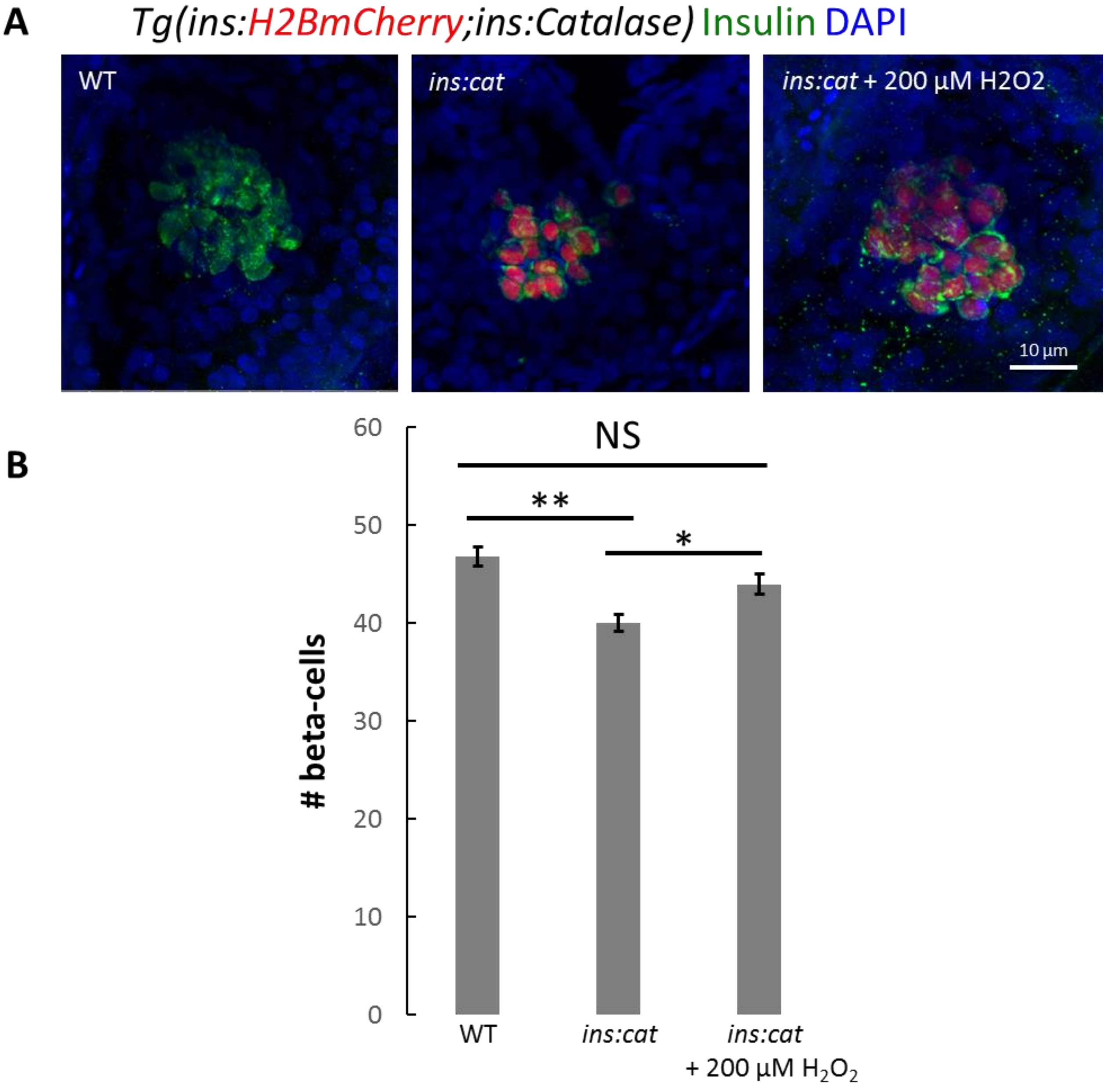

Figure S1: H_2O_2-treatment restores beta-cell number in *Tg(ins:catalase;ins:H2BmCherry)* larvae.
(A) Confocal projections of the principal islets of 4 dpf WT and *Tg(ins:catalase;ins:H2BmCherry)* larvae treated with vehicle or 200 µM H_2O_2 from 2 to 4 dpf. **(B)** Quantification of the average number of beta-cells in WT (n=32), *Tg(ins:catalase;ins:H2BmCherry)* (n=40), and *Tg(ins:catalase;ins:H2BmCherry)* larvae treated with 200µM H_2O_2 (n=40). The average number of beta-cells in *Tg(ins:catalase;ins:H2BmCherry)* animals was significantly reduced compared to WT (p=2.8E-06), whereas in the H_2O_2-treated *Tg(ins:catalase;ins:H2BmCherry)* larvae, this number was not different, indicating that exogenous H_2O_2-administration can restore beta-cell mass. Error bars = SEM. NS = Not significant.

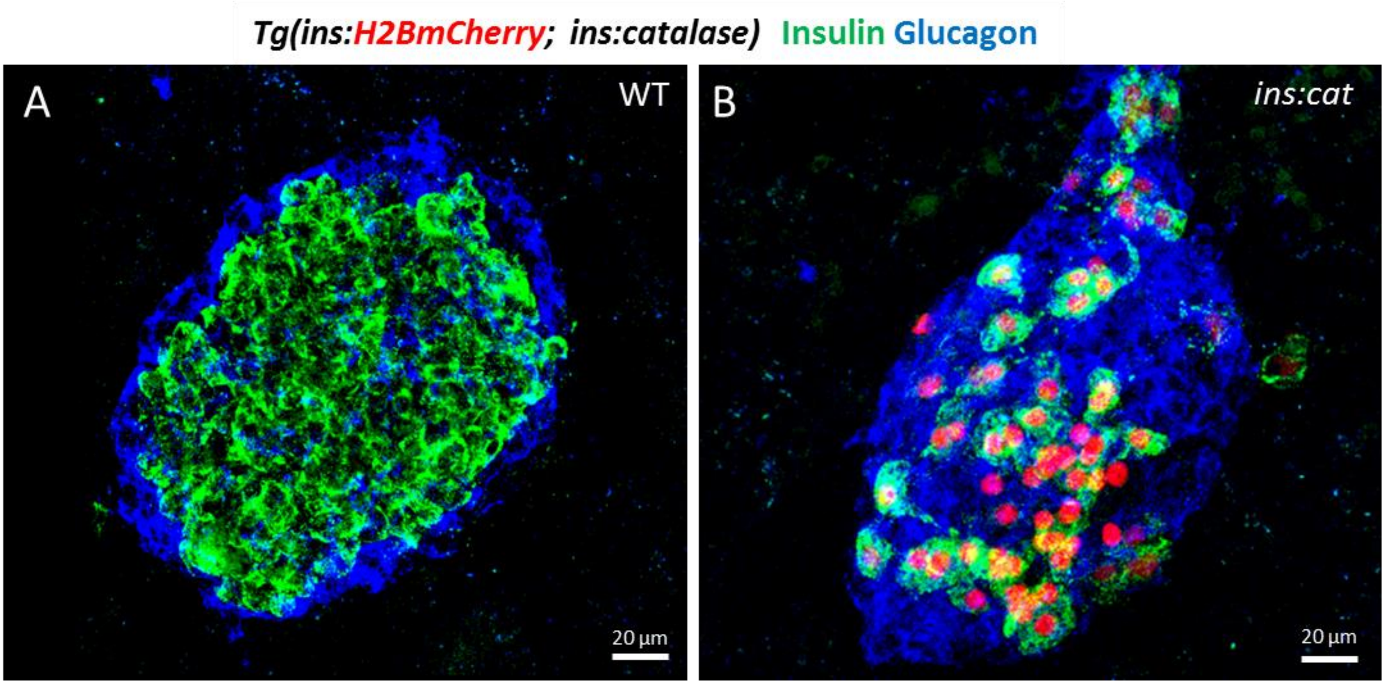

Figure S2: Catalase expression in beta-cells leads to a reduction in beta-cell mass. **(A-B)** Confocal projections spanning the volume of the principal islets of WT and *Tg(ins:catalase;ins:H2BmCherry)* animals stained for insulin and glucagon. The beta-cells in the principal islet of the *Tg(ins:catalase;ins:H2BmCherry)* animals (B) were scattered and fewer in numbers as compared to WT (A).

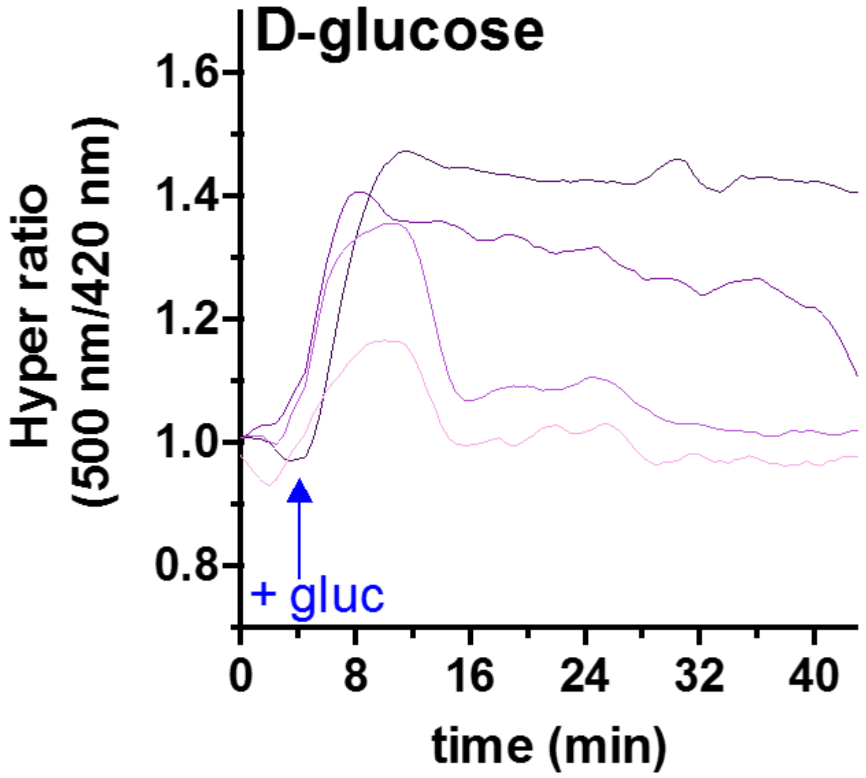

Figure S3: Heterogeneity of H_2O_2 levels in response to glucose.
Quantification of HyPer-3 ratio in INS-1 cells showing distinct dynamics of H_2O_2 levels in response to 18 mM D-glucose. Note, selected representative long-term single cell tracks from experiments from Fig.3b are shown to illustrate transient and more persistent increases in H_2O_2 levels in response to the same glucose stimulus.

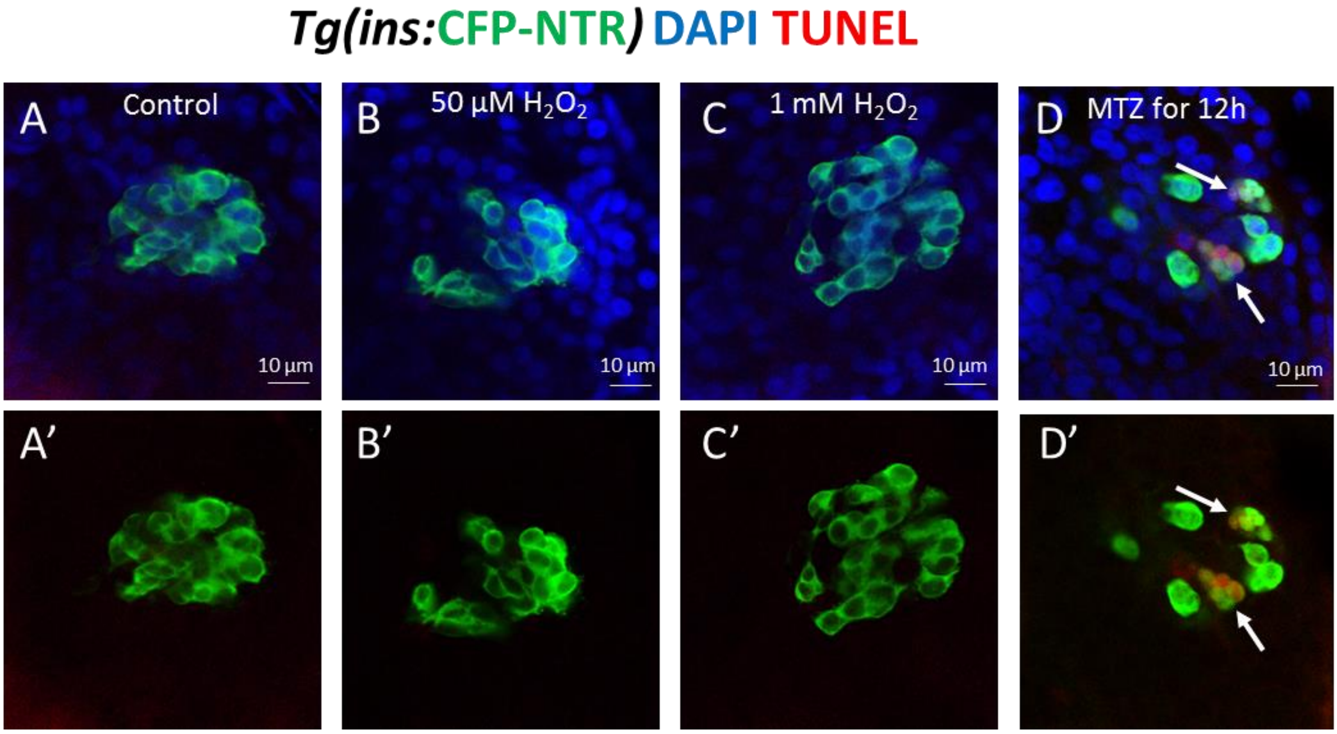

Figure S4: Treatment with different concentrations of H_2O_2 does not cause beta-cell apoptosis. (A-C) Confocal sections of *Tg(ins:CFP-NTR)* larvae. Beta-cells are marker using CFP (green). The larvae were treated with vehicle (A), 50 µM H_2O_2 (B), or 1mM H_2O_2 (C) from 3 to 4 dpf and beta-cell apoptosis was analyzed using the TUNEL assay. No TUNEL-positivity can be observed in the beta-cells or in the surrounding pancreatic tissue. **(D)** *Tg(ins:CFP-NTR)* expresses the Nitroreductase (NTR) enzyme from *Escherichia coli* under the *insulin* promoter. The NTR enzyme converts the pro-drug metronidazole (MTZ) to its cytotoxic form, leading to the specific ablation of beta-cells upon exposure to MTZ. $TUNEL^+$ beta-cells were evident after 12h of treatment with MTZ to induce targeted beta-cell ablation.

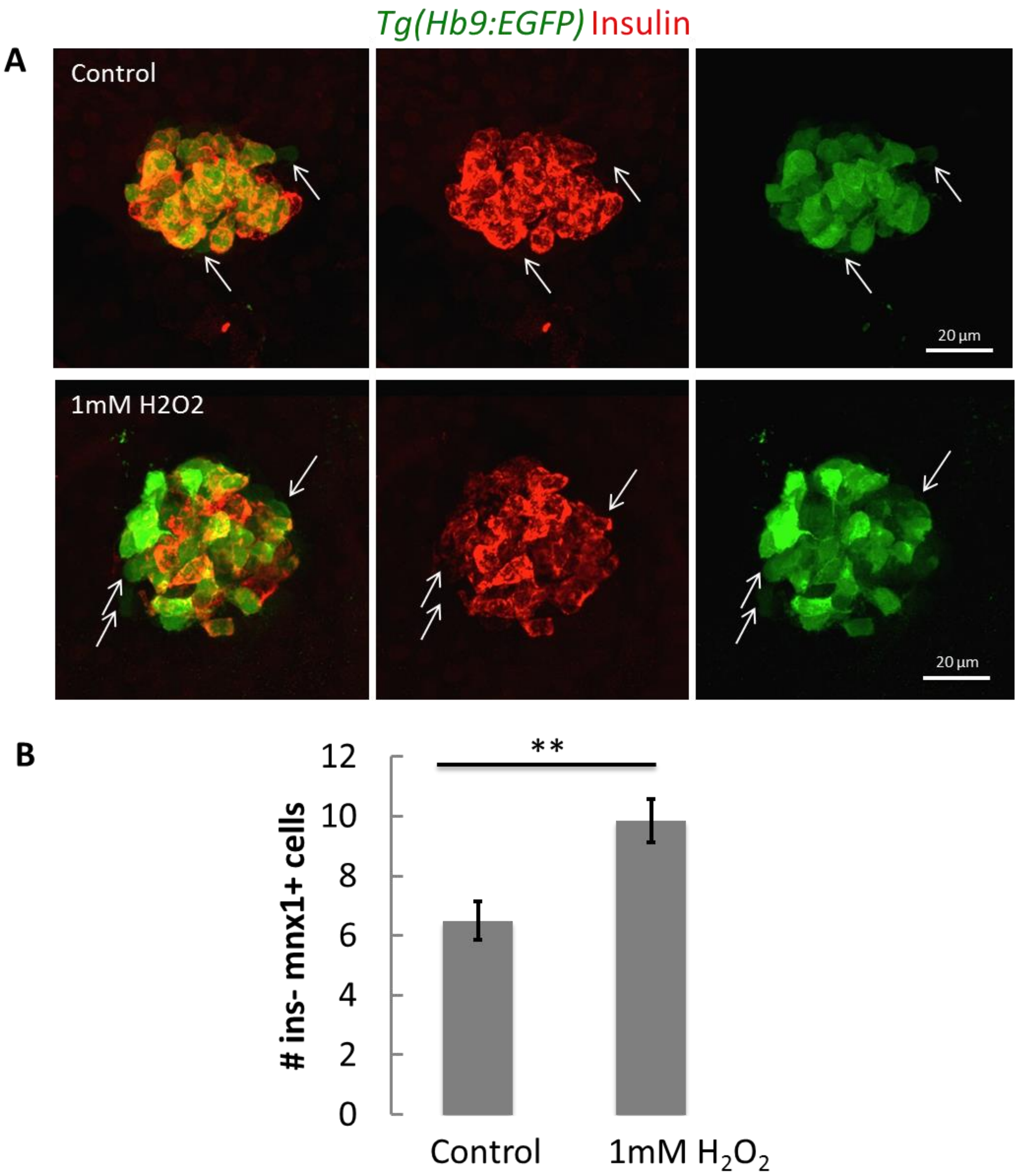

Figure S5: H_2O_2-treatment increases the number of mnx1$^+$/insulin$^-$ cells.

(A) Confocal projections of the principal islets of 4 dpf *Tg(mnx1:GFP)* controls and larvae treated with 1mM H_2O_2 from 3 to 4 dpf. Arrowheads point to *mnx1:*GFP$^+$ but Insulin$^-$ cells. **(B)** Quantification of the number of *mnx1:*GFP$^+$ and insulin$^-$ cells in controls (n=16 animals) and H_2O_2-treated animals (n=13 animals). H_2O_2-treated animals exhibit higher numbers of *mnx1:*GFP$^+$ and Insulin$^-$ cells compared to controls (p= 0,002). Error bars = SEM.

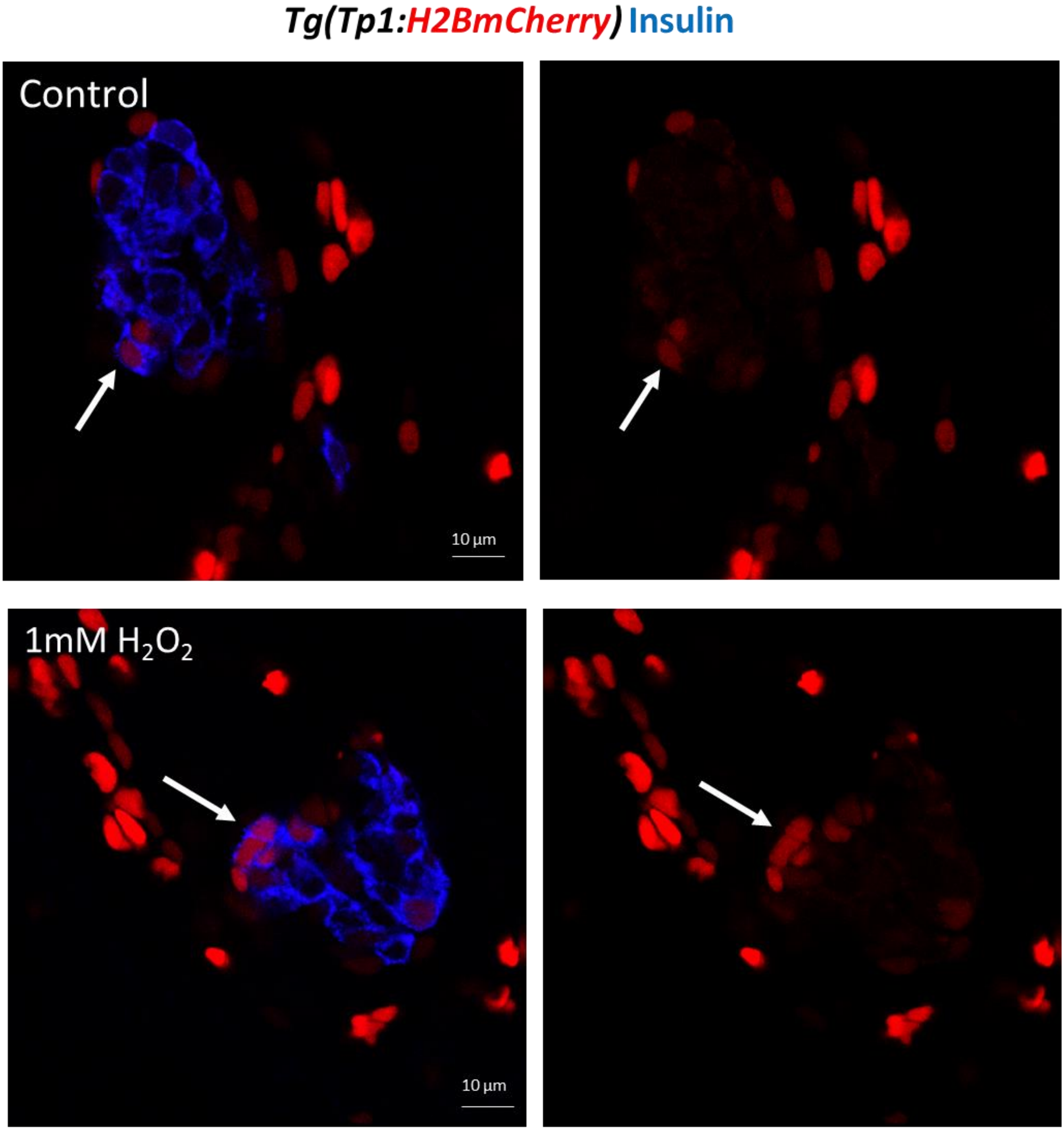

Figure S6: Higher resolution images corresponding to the panels shown in Figure 5D. Confocal sections of primary islets from *Tg(Tp1:H2BmCherry)* larvae stained for Insulin (blue). *Tg(Tp1:H2BmCherry)* drives expression of a fluorescent protein with long half-life (H2BmCherry) in the Notch responsive cells (NRCs) in the pancreas. Beta-cells that differentiate from NRCs can retain H2BmCherry-flurescence due to perdurance. The larvae were treated with vehicle or 1 mM H_2O_2 from 3 to 4 dpf. The arrows indicate *Tp1:*H2BmCherry$^+$ and insulin$^+$ cells in the periphery of the primary islets in controls and H_2O_2-treated larvae.

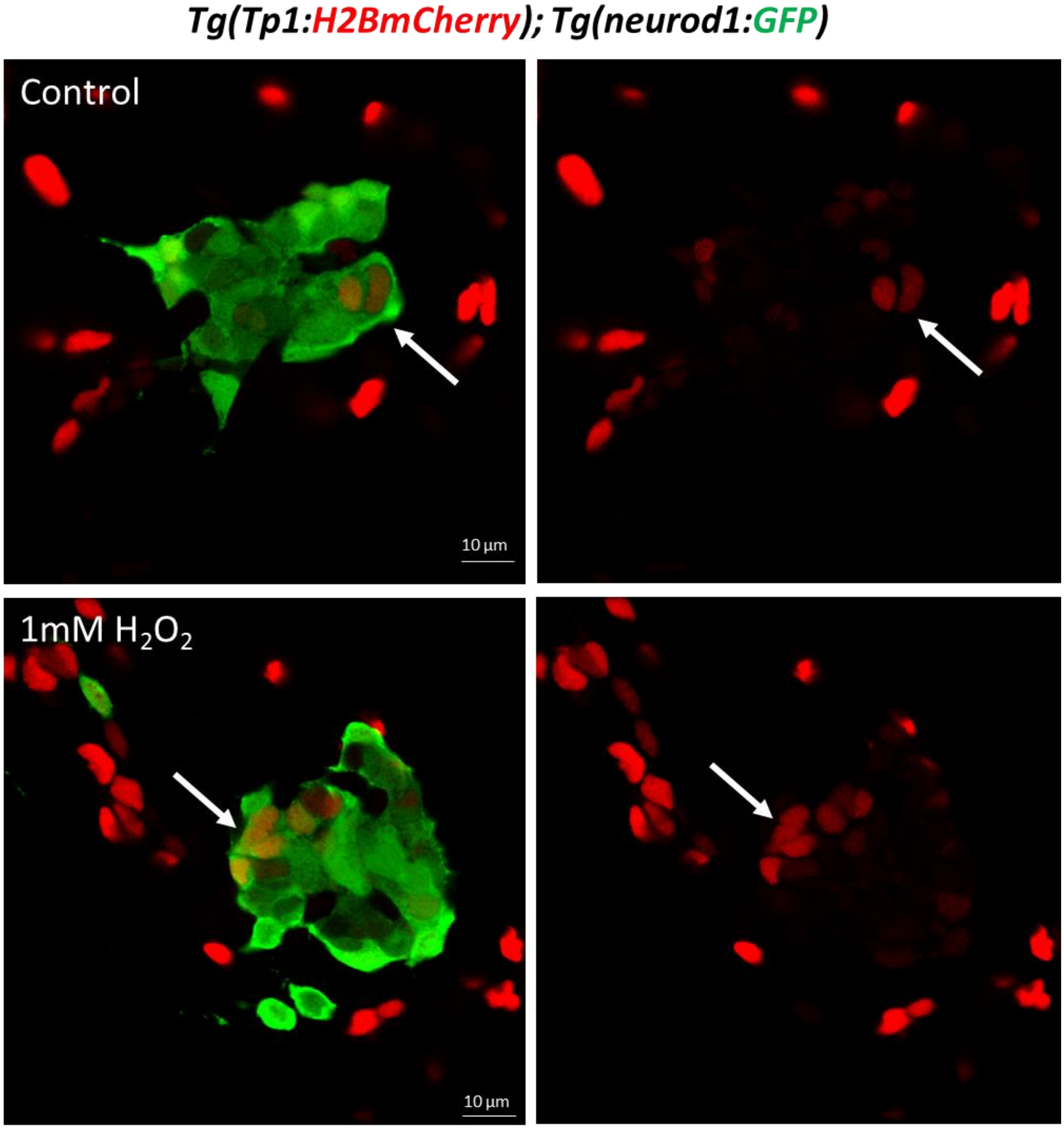

Figure S7: Higher resolution images corresponding to the panels shown in Figure 5F. Confocal sections of primary islets from *Tg(Tp1:H2BmCherry); Tg(neurod1-GFP)* larvae. The arrows indicate *Tp1*:H2BmCherry^{+} and GFP^{+} cells in the periphery of the primary islets in controls and H2O2-treated larvae.
